# Functional Characterization of Secreted Aspartyl Proteases in Candida parapsilosis

**DOI:** 10.1128/mSphere.00484-19

**Published:** 2019-08-21

**Authors:** Dhirendra Kumar Singh, Tibor Németh, Alexandra Papp, Renáta Tóth, Szilvia Lukácsi, Olga Heidingsfeld, Jiri Dostal, Csaba Vágvölgyi, Zsuzsa Bajtay, Mihály Józsi, Attila Gácser

**Affiliations:** a Department of Microbiology, University of Szeged, Szeged, Hungary; b Complement Research Group, Department of Immunology, ELTE Eötvös Loránd University, Budapest, Hungary; c MTA-ELTE Immunology Research Group, Eötvös Loránd University, Budapest, Hungary; d Institute of Organic Chemistry and Biochemistry, Academy of Sciences of the Czech Republic, Prague, Czechia; e Department of Immunology, Eötvös Loránd University, Budapest, Hungary; f Department of Microbiology, University of Szeged Interdisciplinary Excellence Centre, Szeged, Hungary; g MTA-SZTE Lendület Mycobiome Research Group, University of Szeged, Szeged, Hungary; Carnegie Mellon University

**Keywords:** *Candida parapsilosis*, complement, host-pathogen interactions, proteases, virulence

## Abstract

Candida parapsilosis is an emerging non-*albicans Candida* species that largely affects low-birth-weight infants and immunocompromised patients. Fungal pathogenesis is promoted by the dynamic expression of diverse virulence factors, with secreted proteolytic enzymes being linked to the establishment and progression of disease. Although secreted aspartyl proteases (Sap) are critical for Candida albicans pathogenicity, their role in C. parapsilosis is poorly elucidated. In the present study, we aimed to examine the contribution of C. parapsilosis *SAPP* genes *SAPP1*, *SAPP2*, and *SAPP3* to the virulence of the species. Our results indicate that *SAPP1* and *SAPP2*, but not *SAPP3*, influence adhesion, host cell damage, phagosome-lysosome maturation, phagocytosis, killing capacity, and cytokine secretion by human peripheral blood-derived macrophages. Purified Sapp1p and Sapp2p were also shown to efficiently cleave host complement component 3b (C3b) and C4b proteins and complement regulator factor H. Additionally, Sapp2p was able to cleave factor H-related protein 5 (FHR-5). Altogether, these data demonstrate the diverse, significant contributions that *SAPP1* and *SAPP2* make to the establishment and progression of disease by C. parapsilosis through enabling the attachment of the yeast cells to mammalian cells and modulating macrophage biology and disruption of the complement cascade.

**IMPORTANCE** Aspartyl proteases are present in various organisms and, among virulent species, are considered major virulence factors. Host tissue and cell damage, hijacking of immune responses, and hiding from innate immune cells are the most common behaviors of fungal secreted proteases enabling pathogen survival and invasion. C. parapsilosis, an opportunistic human-pathogenic fungus mainly threatening low-birth weight neonates and children, possesses three *SAPP* protein-encoding genes that could contribute to the invasiveness of the species. Our results suggest that *SAPP1* and *SAPP2*, but not *SAPP3*, influence host evasion by regulating cell damage, phagocytosis, phagosome-lysosome maturation, killing, and cytokine secretion. Furthermore, *SAPP1* and *SAPP2* also effectively contribute to complement evasion.

## Abstract (precis)

Aspartyl proteases are present in various organisms and, among virulent species, are considered major virulence factors. Host tissue and cell damage, hijacking of immune responses, and hiding from innate immune cells are the most common behaviors of fungal secreted proteases enabling pathogen survival and invasion. C. parapsilosis, an opportunistic human-pathogenic fungus mainly threatening low-birth weight neonates and children, possesses three *SAPP* protein-encoding genes that could contribute to the invasiveness of the species. Our results suggest that *SAPP1* and *SAPP2*, but not *SAPP3*, influence host evasion by regulating cell damage, phagocytosis, phagosome-lysosome maturation, killing, and cytokine secretion. Furthermore, *SAPP1* and *SAPP2* also effectively contribute to complement evasion.

## INTRODUCTION

*Candida* infections are associated with a high socioeconomic impact and with morbidity and mortality among infants, children, and the elderly worldwide (1, 2). Among the non*-albicans* species, the incidence of infections caused by Candida parapsilosis is increasing worldwide and C. parapsilosis is currently the second or third most common yeast species associated with invasive candidiasis in hospitals in Asian, European, and South American countries (3). C. parapsilosis is commonly associated with low-birth-weight neonate infections, invasive infections of hospitalized immunocompromised patients, and the receipt of parenteral nutrition or prolonged use of intravascular devices (4). Despite its clinical significance, the pathogenicity of C. parapsilosis and its virulence factors and interactions with the host are still poorly understood (5–7).

Aspartyl proteases are present in various organisms and are most active at acidic pH (pH 1.9 to 4.0), share a catalytic apparatus, and cleave dipeptide bonds between two hydrophobic amino acid residues (8). Fungal secreted aspartyl proteases are reported to directly mediate virulence (9–13). C. parapsilosis possesses three aspartyl acid protease-encoding genes, namely, *SAPP1*, *SAPP2*, and *SAPP3*. *SAPP1* is duplicated in the species’ genome (*SAPP1a*, *SAPP1b*) (14). A previously established Δ/Δ*sapp1a* Δ/Δ*sapp1b* strain, lacking *SAPP1*, was shown to be hypersusceptible to human serum (HS), caused attenuated host cell damage, and was phagocytosed and killed more efficiently by human monocytes and macrophages than the wild-type strain (15). In another study using reconstituted human oral epithelium (RHOE), levels of tissue damage caused by C. parapsilosis were significantly reduced in the presence of the Sapp inhibitor pepstatin, further highlighting the role of secreted proteases in the species’ pathogenicity (16).

Upon superficial infection, epithelial cells trigger an inflammatory response by producing antimicrobial peptides and recruiting and activating innate immune cells, including macrophages and neutrophils (17–19). *Candida* species can efficiently avoid macrophage-mediated killing by host membrane rupture, secretion of proteases and lipases, and induction of pyroptosis and by nutrient competition with the host (20–22). Upon infection, the complement cascade also activates and plays a role in combating pathogens via enhancing chemotaxis, phagocytosis, or T and B cell differentiation (23). Pathogenic species have adopted several strategies to evade complement attack (24). In particular, C. albicans either recruits complement regulator proteins on its surface or cleaves complement proteins by secreting the proteases. C. parapsilosis can also bind to human complement proteins; however, the effect of this binding has not been fully resolved (25, 26).

To date, multiple studies have shown that C. albicans aspartyl proteases have different abilities to damage epithelial cells, alter the host complement cascade, induce macrophage chemotaxis or cytokine production, and mediate NLRP3 inflammasome activation; less is known about the immune modulatory effects of aspartyl proteases in C. parapsilosis (12, 27). Therefore, to elucidate the role of individual aspartyl proteases in the virulence of C. parapsilosis, *SAPP* mutant strains were generated. Functional characterization of these genes revealed that *SAPP1* and *SAPP2* (but not *SAPP3*) play an important role in C. parapsilosis pathogenicity.

## RESULTS

### Generation and characterization of *RI_SAPP1*, *RI_SAPP2*, and *RI_SAPP3* strains.

Aspartyl protease-encoding genes in C. albicans are associated with various physiological and pathogenic roles. For instance, expression of SAPI to SAPIII has been associated with the yeast form of this species and linked with phenotypic switching. Previously, high levels of expression of SAPIV *to* SAPVI have been associated with the hyphal phase, suggesting their assistance in pathogenicity development; however, their involvement in virulence regulation is still debatable (14, 28, 29). The precise role of these genes in virulence in C. parapsilosis is not well studied. Therefore, we sought to expand upon prior work to further evaluate the biology of C. parapsilosis *SAPP1* and to robustly characterize the function of *SAPP2* and *SAPP3*. To delineate the roles of C. parapsilosis aspartyl proteases in virulence, we aimed to overexpress *SAPP1*, *SAPP2*, and *SAPP3* genes individually under the control of a constitutive promoter (*CaTDH3*), integrated into the C. parapsilosis neutral locus (*CpNEUT5L*) of the *SAPP1*-*SAPP2*-*SAPP3* (*sapp1*/*2*/*3^−^*^/^*^−^*) null mutant strain.

All reintegrant mutant strains were established on the *sapp1*/*2*/*3^−^*^/^*^−^* background to avoid cross-interference from each Sapp. Mutant strains were confirmed by colony PCR and Southern blotting (data not shown).

**FIG 1:**
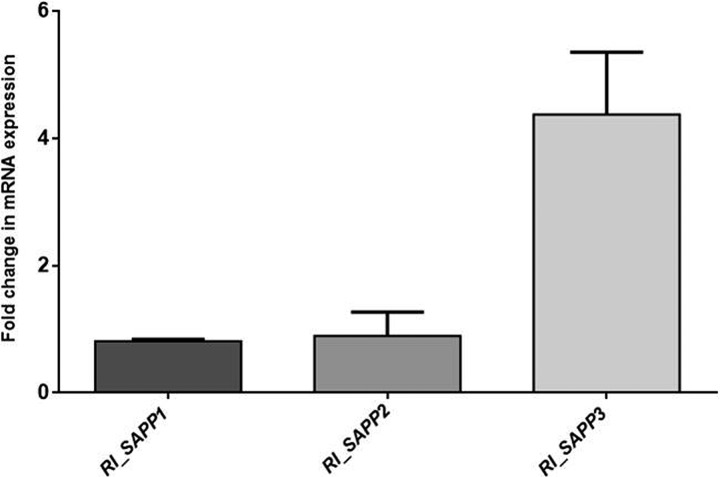
mRNA expression of *RI_SAPP1*, *RI_SAPP2*, and *RI_SAPP3* strains. Data represent fold changes in expression levels of *SAPP* genes in *RI_SAPP* mutants compared to the corresponding genes in the wild-type strain after growth in YCB plus 0.2% BSA medium. The figure represents data obtained from triplicate experiments.

Expression levels of *SAPP* genes in the reintegrant mutant strains were determined using real-time PCR. Wild-type and mutant strains were cultivated in secreted-protease-inducing medium (yeast carbon base [YCB] plus 0.2% bovine serum albumin [BSA]), and the levels of expression of *SAPP1*, *SAPP2*, and *SAPP3* were monitored after 48 h of incubation. The levels of expression of genes *SAPP1* and *SAPP2* in reintegrant strains *RI_SAPP1* and *RI_SAPP2* were similar to what was observed in the wild-type strains, while the level of expression of *SAPP3* was upregulated in the *RI_SAPP3* strain by ≥4-fold (Fig. 1).

Next, we examined whether reintegration of *SAPP* genes altered the viability, morphology, or biofilm-forming ability of the mutant strains. No difference was observed between the levels of growth of the mutants in either yeast extract-peptone-dextrose (YPD) or YCB liquid medium at 30°C and the levels seen with the wild-type strain (see Fig. S1A and B in the supplemental material), and the *SAPP* mutant strains produced elongated pseudohyphae to the same extent as the reference strain in YPD or RPMI medium supplemented with 10% fetal bovine serum (FBS) and spider liquid medium (Fig. S2A to C). We observed no difference in colony morphologies (Fig. S3) or in biofilm-forming abilities (Fig. S4). We also tested the ability of the *sapp1*/*2*/*3^−^*^/^*^−^* mutant to cope with stress by monitoring cell growth in the presence of several stressors (Table S3). The *sapp1*/*2*/*3^−^*^/^*^−^* mutant strain showed no differences in growth in the presence of stressors (Fig. S5). These results demonstrate that the mutant strains retained the physiological attributes and stress responses of the parental strain.

10.1128/mSphere.00484-19.1 FIG S1 Growth kinetics of the wild-type and mutant strains. Data represent levels of growth of the wild-type and mutant strains in YPD (A) and in YCB plus BSA medium (B). Strains were grown at 30°C, and the optical density (OD) at 600 nm was measured every 30 min for 24 h. Download FIG S1, TIF file, 0.5 MB. Copyright © 2019 Singh et al. 2019 Singh et al. This content is distributed under the terms of the Creative Commons Attribution 4.0 International license.

10.1128/mSphere.00484-19.2 FIG S2 Pseudohypha formation by C. parapsilosis wild-type and mutant strains. (A) Representative images of C. parapsilosis wild-type and mutant strains stained with concanavalin A-fluorescein isothiocyanate (ConA-FITC). (B) Percentage of cells forming pseudohyphae based on manual image analysis of microscopic pictures. (C) Percentage of cells forming pseudohyphae analyzed by flow cytometry. Bar, 20 μm. Download FIG S2, TIF file, 1.4 MB. Copyright © 2019 Singh et al. 2019 Singh et al. This content is distributed under the terms of the Creative Commons Attribution 4.0 International license.

10.1128/mSphere.00484-19.3 FIG S3 Deletion and overexpression of the *SAPP* gene in C. parapsilosis did not alter morphology. Morphology was examined after growth of wild-type and mutant strains on YPD and spider agaric plates for 5 days at 37 and 30°C. Download FIG S3, TIF file, 0.7 MB. Copyright © 2019 Singh et al. 2019 Singh et al. This content is distributed under the terms of the Creative Commons Attribution 4.0 International license.

10.1128/mSphere.00484-19.4 FIG S4 Biofilm formation. Biofilm levels were quantified by crystal violet (A) and 3-(4,5-dimethyl-2-thiazolyl)-2,5-diphenyl-2H-tetrazolium bromide (MTT) (B) assays. C. parapsilosis wild-type and mutant strains formed biofilm after growth either in RPMI or in yeast nitrogen base (YNB) media. Download FIG S4, TIF file, 0.2 MB. Copyright © 2019 Singh et al. 2019 Singh et al. This content is distributed under the terms of the Creative Commons Attribution 4.0 International license.

10.1128/mSphere.00484-19.5 FIG S5 Phenotypical characterization of C. parapsilosis wild-type and *sapp1*/*2*/*3^−^*^/^*^−^* strains. Strains grown overnight in YPD were serially diluted, and 5-μl volumes of the dilutions containing 10^4^, 10^3^, 10^2^, and 10 cells were plated on YPD plates without or with the addition of stress-inducing reagents (described in Materials and Methods) and grown at 30°C. Download FIG S5, TIF file, 0.8 MB. Copyright © 2019 Singh et al. 2019 Singh et al. This content is distributed under the terms of the Creative Commons Attribution 4.0 International license.

### Semiquantitative detection of extracellular protease activity of *SAPP* mutant strains.

**FIG 2:**
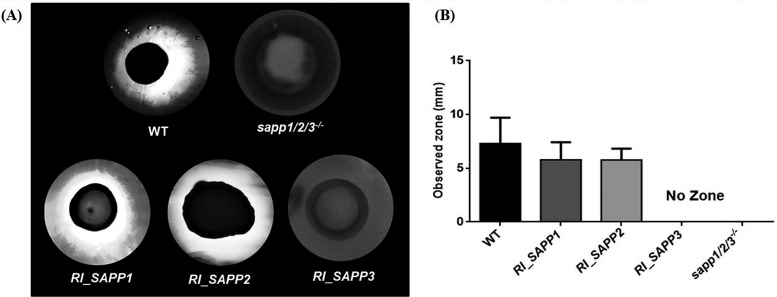
The protease activity of wild-type, *sapp1*/*2*/*3^−^*^/^*^−^*, and *RI_SAPP* strains was examined by BSA degradation assay. (A) A total of 10^6^ *Candida* cells were spotted on YCB plus 0.2% BSA solid plates and incubated at 30°C for 3 days. The width of the proteolytic halo zone was determined by amido black staining. Images are representative of results from 3 independent repeated experiments. WT, wild type. (B) The radius (in millimeters) of each clearance (or proteolytic) zone was also measured.

*Candida* secreted aspartyl proteases hydrolyze BSA present in agar plates. In order to examine the secreted protease activity of the established strains, the wild-type and *SAPP* mutant strains were spotted on plates containing YCB plus 0.2% BSA and, following amido black staining, the width of the clearance zone was measured. The C. parapsilosis wild-type strain showed a clear halo zone (7.3 mm in diameter) on BSA-containing plates similar to the zones seen with strains *RI_SAPP1* (5.78 mm) and *RI_SAPP2* (5.76 mm). The *RI_SAPP3* and *sapp1*/*2*/*3^−^*^/^*^−^* strains, however, showed no proteolytic activity (Fig. 2). These results suggest that, in contrast to *SAPP1* and *SAPP2*, reintegration of *SAPP3* does not restore the aspartyl protease activity of the *sapp1*/*2*/*3^−^*^/^*^−^* strain; thus, *SAPP3* does not contribute to aspartyl protease secretion in this species.

### C. parapsilosis *RI_SAPP3* and *sapp1*/*2*/*3^−^*^/^*^−^* strains are sensitive to human serum.

**FIG 3:**
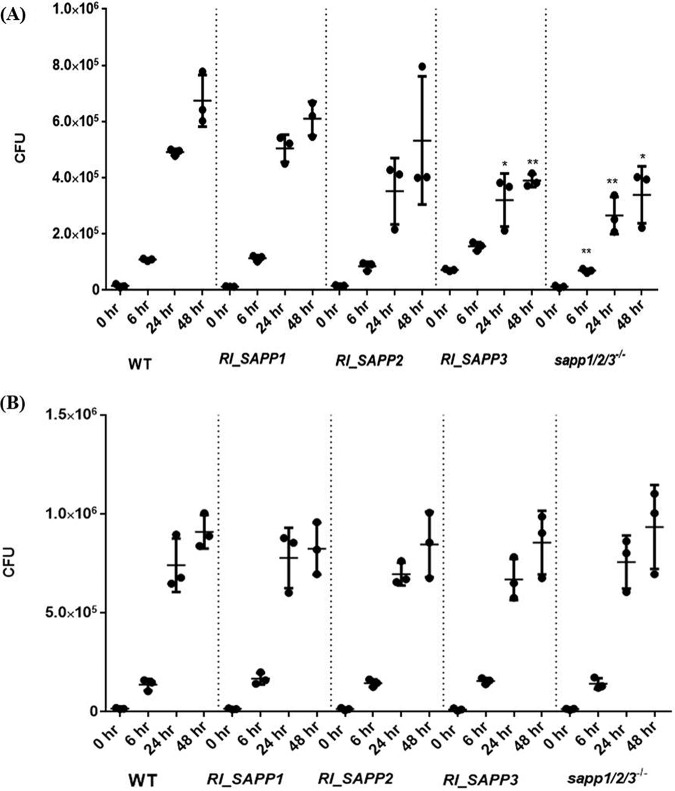
Serum sensitivity assay. The growth of C. parapsilosis wild-type and mutant strains in 20% NHS (A) and HiS (B) was examined by determination of CFUs at 0, 6, 24, and 48 h. Data were obtained from three independent experiments. Differences between groups were considered statistically significant at *P* < 0.05. *, *P* < 0.05; **, *P* < 0.01.

To investigate the fungicidal effect of human serum on the examined strains, yeast cells were cultivated in the presence of normal human serum (NHS) and CFU determinations were performed at different time intervals. C. parapsilosis strains were also grown in the presence of 20% heat-inactivated serum (HiS). The viability of the *RI_SAPP3* and *sapp1*/*2*/*3^−^*^/^*^−^* strains was reduced significantly after 18 and 24 h of incubation in intact serum compared to the wild-type strain results, while the *RI_SAPP1* and as *RI_SAPP2* strains showed no sensitivity to NHS (Fig. 3A). However, no sensitivity was observed after HiS treatment (Fig. 3B). These data suggest that Sapp1 and Sapp2 are involved in protection against human serum proteins but that Sapp3 is not associated with this effect.

### Secreted aspartyl proteases affect the adhesion capabilities of C. parapsilosis.

**FIG 4:**
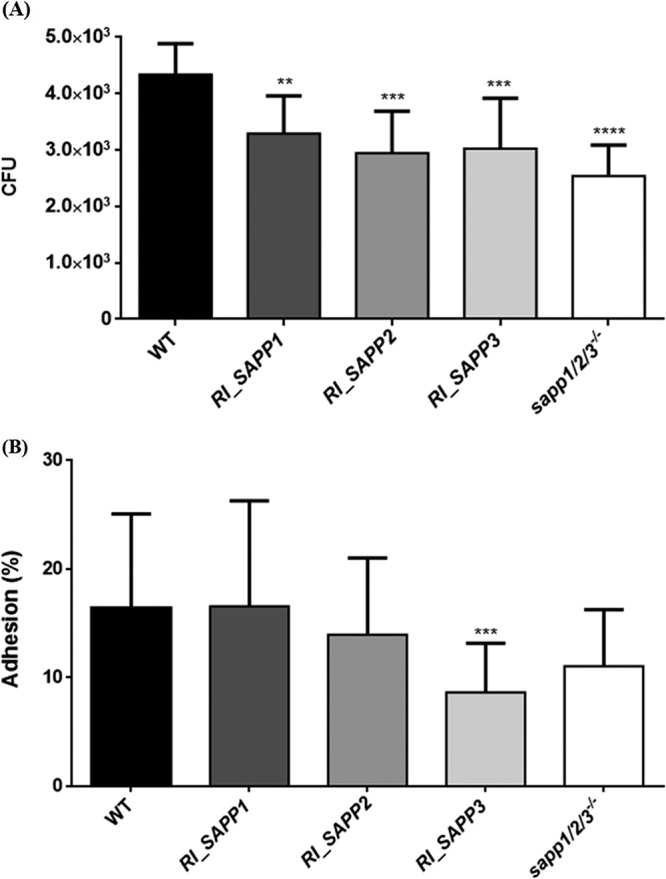
*In vitro* cell adhesion assay. The ability of the wild-type and mutant strains to adhere to polystyrene surfaces (A) and to TR146 epithelial cells (B) was assayed. Results (means ± standard errors of the means [SEM]) were gained from at least three independent experiments. *, *P* < 0.05; **, *P* < 0.01; ***, *P* < 0.002; ****, *P* < 0.0001.

We further examined whether *SAPP* genes influence the adhesion properties of C. parapsilosis by the use of biotic and abiotic surfaces. Results of the cell adhesion assays showed that all three reintegrated mutant strains had significantly reduced capabilities of adhesion to polystyrene surfaces compared to the reference strain (Fig. 4A). The highest reduction in adhesion was observed with the *sapp1*/*2*/*3^−^*^/^*^−^* strain (approximately 40%), followed by *RI_SAPP2* (25%), *RI_SAPP3* (25%), and the *RI_SAPP1* strain (20%).

A significant reduction in adhesion to cells of the TR146 human oral epithelial cell line was observed with strain *RI_SAPP3*, while a moderate decrease was detected in the case of the *sapp1*/*2*/*3^−^*^/^*^−^* strain (Fig. 4B).

### *SAPP1* and *SAPP2* partially restore the damage-causing capability of the *sapp1*/*2*/*3^−^*^/^*^−^* strain.

**FIG 5:**
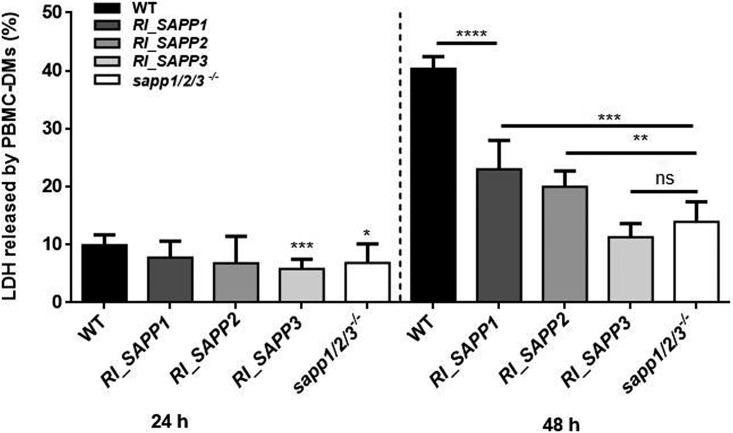
Damage-causing efficiency of wild-type and mutant strains by LDH release. Human PBMC-DMs were infected with the C. parapsilosis wild-type strain or mutant strain *RI_SAPP* or *sapp1*/*2*/*3^−^*^/^*^−^* for 24 and 48 h, and levels of LDH release were measured. The obtained data represent macrophages obtained from six healthy donors. *, *P* < 0.05; **, *P* < 0.01; ***, *P* < 0.002; ****, *P* < 0.0001; ns, not significant.

The ability of the wild-type, *SAPP* mutant, and *sapp1*/*2*/*3^−^*^/^*^−^* strains to damage peripheral blood mononuclear cell-derived macrophages (PBMC-DMs) was monitored by lactate dehydrogenase (LDH) release 24 and 48 h after coincubation. As shown in Fig. 5, the wild-type, *RI_SAPP1*, and *RI_SAPP2* strains induced levels of damage similar to those seen with PBMC-DMs (7.779% ± 1.001% and 6.807% ± 1.642%, respectively), whereas the *RI_SAPP3* and *sapp1*/*2*/*3^−^*^/^*^−^* strains caused significantly less damage (5.843% ± 0.5715% and 6.862% ± 1.340%, respectively) than the wild-type strain (9.944% ± 0.6143%) after 24 h of coincubation. Differences between the examined strains became more evident following 48 h of coincubation. Host cell damage was least severe in macrophages infected with the *RI_SAPP3* and *sapp1*/*2*/*3^−^*^/^*^−^* strains (11.28% ± 0.8304% and 13.95% ± 1.153%, respectively), followed by *RI_SAPP2* (19.98% ± 1.238%) and *RI_SAPP1* (23.04% ± 1.661), compared to that seen with the wild-type strain (40.36% ± 0.6912%) (Fig. 5). These results suggest that *SAPP1* and *SAPP2* (but not *SAPP3*) contribute to the killing of PBMC-DMs.

### Macrophages phagocytose and kill *RI_SAPP3* and *sapp1*/*2*/*3^−^*^/^*^−^* mutants more efficiently than wild-type and *RI_SAPP1* and *RI_SAPP2* cells.

**FIG 6:**
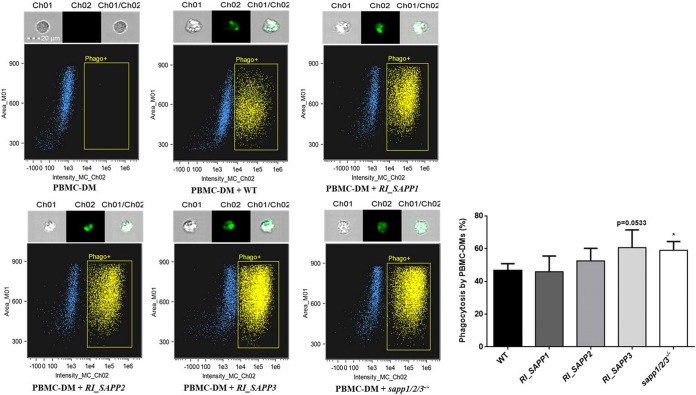
Phagocytosis of wild-type and *RI_SAPP* and *sapp1*/*2*/*3^−^*^/^*^−^* mutant strains by human-blood-derived macrophages determined by flow cytometry. Human PBMC-DMs were coincubated individually with Alexa Fluor 488-labeled fungal strains at 37°C for 2 h. Fungal cell-containing macrophages (phago+) were identified by flow-cytometry and the percentage of phagocytosis was determined. Data were obtained from five independent experiments. *, *P* < 0.05.

**FIG 7:**
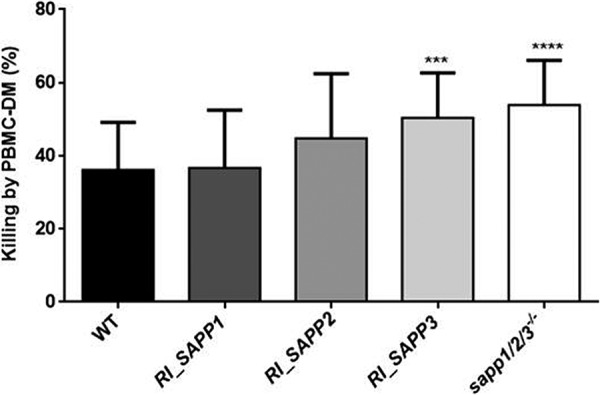
Killing of C. parapsilosis strains by human PBMC-DMs. Human PBMC-DMs were coincubated with C. parapsilosis wild-type and *sapp* mutant strains at 37°C for 3 h, and levels of yeast killing efficiency were determined by CFU counting. Data were obtained using four healthy donors. *, *P* < 0.05; **, *P* < 0.01; ***, *P* < 0.002.

We first examined the phagocytic capacity of PBMC-DMs by fluorescence-activated cell sorter (FACS) analysis. Yeast cells were labeled with the fluorescent dye Alexa Fluor 488 and coincubated with PBMC-DMs for 2 h at 37°C in the presence of 5% CO_2_. Our results indicated that PBMC-DMs ingested *RI_SAPP3* and *sapp1*/*2*/*3^−^*^/^*^−^* more efficiently than the wild-type strain (Fig. 6). We also examined the yeast cell killing efficiency of PBMC-DMs by comparing the recovered fungal CFU counts after coincubation. Our data showed that PBMC-DMs were able to kill significantly more *RI_SAPP3* (50.39% ± 2.328%) and *sapp1*/*2*/*3^−^*^/^*^−^* (53.90% ± 2.262%) cells than the wild-type strain (36.14% ± 2.652%) and strains *RI_SAPP1* (36.72% ± 2.930%) and *RI_SAPP2* (44.82% ± 3.598%) (Fig. 7).

### Aspartyl proteases promote intracellular survival of C. parapsilosis by altering phagosome-lysosome maturation.

**FIG 8:**
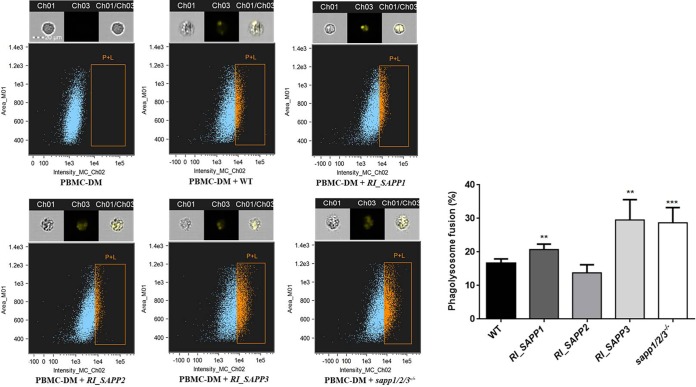
Phagosome-lysosome fusion following the uptake of wild-type and *SAPP* mutants. PBMC-DMs were infected with yeast cells labeled with pHrodo at a 1:5 ratio and were then incubated at 37°C for 2 h. Phagosome-lysosome fusion was then determined by flow cytometry. Ch1, bright-field image; Ch3, green fluorescence channel; Ch1/Ch3, merged image. Graph showing the extent of phagosome-lysosome fusion for the wild-type and mutant strains. *n* = 5. **, *P* < 0.01; ***, *P* < 0.002.

A previous study reported that *Candida* cells can replicate and survive within macrophages, either by diverting the normal process of phagosome maturation, causing physical damage, or by withstanding the hostile environment of the mature phagosome-lysosome (30). Here, we aimed to examine if C. parapsilosis aspartyl proteases influence phagosome-lysosome maturation in human PBMC-DMs. We analyzed the phagosome-lysosome maturation after coincubating pHrodo-stained *Candida* cells with PBMC-DMs for 2 h. Interestingly, PBMC-DMs infected with the wild-type strain, mutant strain *RI_SAPP1*, and mutant strain *RI_SAPP2* showed a lower rate of phagosome-lysosome fusion (16.66% ± 0.5732%, 20.76% ± 0.7194%, and 13.78% ± 1.216%, respectively) than was seen with *RI_SAPP3* (29.52% ± 2.719%) and *sapp1*/*2*/*3^−^*^/^*^−^* (28.70% ± 2.025%), indicating that Sapp1 and Sapp2 (but not Sapp3) may promote intracellular survival of C. parapsilosis in human macrophages (Fig. 8).

### C. parapsilosis Sapp proteins regulate the cytokine response of host macrophages.

**FIG 9:**
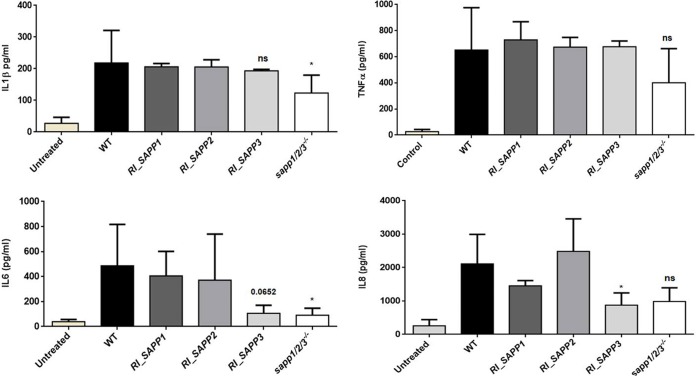
Cytokine secretion by human-blood-derived macrophages in response to wild-type and *SAPP* mutant strains. Levels of IL-1β (upper left panel), TNF-α (upper right panel), IL-6 (lower left panel), and IL-8 (lower right panel) were measured by ELISA after stimulation of PBMC-DMs with the wild-type strain or a *SAPP* mutant strain for 24 h. Data represent levels of cytokine production by macrophages obtained from 5 healthy donors. *, *P* < 0.05.

In order to examine if the cytokine responses triggered by the wild-type strain, the *RI_SAPP* mutants, and strains *sapp1*/*2*/*3^−^*^/^*^−^* differed significantly, we stimulated human PBMC-DMs for 24 h with each strain and measured interleukin-1β (IL-1β), tumor necrosis factor alpha (TNF-α), IL-6, and IL-8 responses. The obtained results indicated that PBMC-DMs stimulated with either the wild-type strain or the *RI_SAPP1* and *RI_SAPP2* strains produced similar IL-1β, IL-8, and TNF-α levels. In contrast, macrophages stimulated with strain *sapp1*/*2*/*3^−^*^/^*^−^* produced significantly less IL-1β and IL-6 and moderately but not significantly less IL-8 than the wild-type strain (Fig. 9). PBMC-DMs stimulated with *RI_SAPP3* produced significantly lower IL-8 and moderately low IL-6 levels; however, no significant differences were observed in the production of IL-1β and TNF-α compared to wild type.

### Sapp1p and Sapp2p have differential cleavage capacities against human complement proteins.

**FIG 10:**
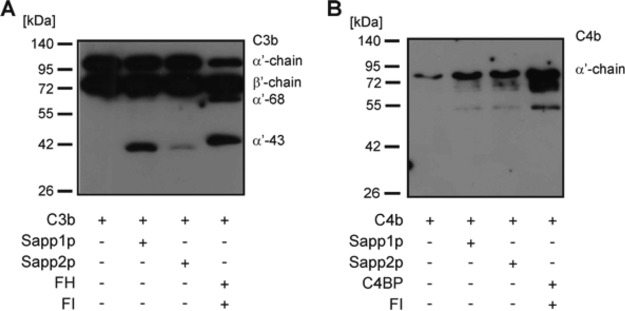
Sapp-mediated cleavage of human complement components C3b and C4b. Sapp1p and Sapp2p were incubated with the main opsonic human complement proteins C3b and C4b. After incubation, the mixture was separated by SDS-PAGE and cleaved C3b (A) and C4b (B) fragments were identified by Western blotting.

**FIG 11:**
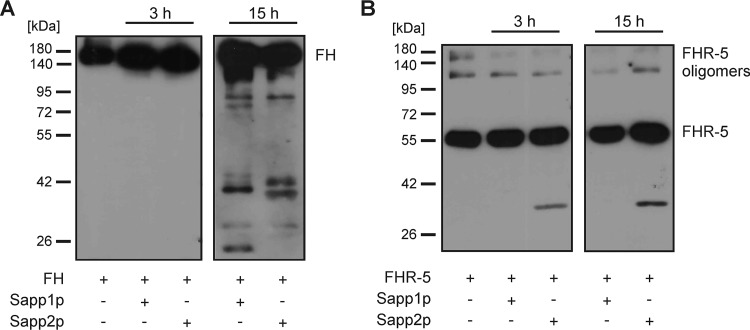
Sapp-mediated cleavage of human complement regulators. Cleavage of FH (A) and FHR-5 (B) by Sapp1p and Sapp2p was determined after 3h and 15 h of incubation.

C. albicans secreted aspartyl proteases can cleave components of human serum, including complement proteins (such as complement component 3b [C3b], C4b, and C5 and the complement regulator FH) and other microbicidal plasma proteins (31, 32). Therefore, to test if C. parapsilosis Sapp proteins are also able to cleave human complement proteins, we incubated C3b and C4b and complement regulatory proteins with the purified Sapp proteins. Our results indicated that the cleavage efficiency of Sapp1p against C3b was higher (shown with stronger cleavage fragment) than that of Sapp2p, which may suggest a difference in the substrate preferences of the two proteases (Fig. 10A). Moreover, Sapp1p and Sapp2p were also able to cleave human C4b (Fig. 10B). Purified C3b and C4b were incubated without Sapp proteins for the same 3-h time period and used as negative controls; cleavage of C3b and cleavage of C4b by factor I in the presence of its cofactors were included as positive controls. To investigate if C. parapsilosis Sapp1p and Sapp2p can cleave complement regulators of the FH protein family, we measured the capacity of Sapp1p and Sapp2p to degrade FH, FHL-1, FHR-1, and FHR-5. Coincubation of Sapp1p or Sapp2p with FHL-1 or FHR-1 revealed that the proteases were not able to cleave these human complement proteins, as visualized by Western blotting (Fig. S6). However, FH was cleaved by both fungal proteases after 15 h of incubation. Interestingly, Sapp2p but not Sapp1p was able to cleave FHR-5 at the early time point of 3 h, further indicating a difference in the substrate preferences of C. parapsilosis Sapp proteins (Fig. 11).

10.1128/mSphere.00484-19.6 FIG S6 Analysis of Sapp-mediated cleavage of human complement regulator proteins. (A and B) Factor H-like protein 1 (FHL-1) and factor H-related protein 1 (FHR-1) were incubated with Sapp1p and Sapp2p for 3 h and 15 h, as indicated. No cleavage of FHL-1 and FHR-1 was detected. Download FIG S6, TIF file, 1.6 MB. Copyright © 2019 Singh et al. 2019 Singh et al. This content is distributed under the terms of the Creative Commons Attribution 4.0 International license.

Since attachment of opsonic complement proteins to pathogens enhances CR3-mediated phagocytosis by macrophages and C. albicans cleaves CR3 and CR4 on macrophages (31), we also tested whether C. parapsilosis Sapp1p and Sapp2p can cleave complement receptors CR3 and CR4; however, we did not find substantial differences in the levels of expression of CR3 and CR4 receptors on macrophages after protease treatment (Fig. S7).

10.1128/mSphere.00484-19.7 FIG S7 Sapp proteins of C. parapsilosis are not able to inactivate CR3 (CD11b/CD18) and CR4 (CD11c/CD18). The levels of expression of CD11b (A and B), CD11c (C and D), and CD18 (E and F) were measured using flow cytometry with monoclonal antibodies (MAbs) specific to these receptor chains. Representative histograms and means ± standard deviations (SD) of mean fluorescence intensity (MFI) values determined for 3 independent donors are shown. Download FIG S7, TIF file, 0.7 MB. Copyright © 2019 Singh et al. 2019 Singh et al. This content is distributed under the terms of the Creative Commons Attribution 4.0 International license.

### Fungal burden and Galleria mellonella survival.

**FIG 12:**
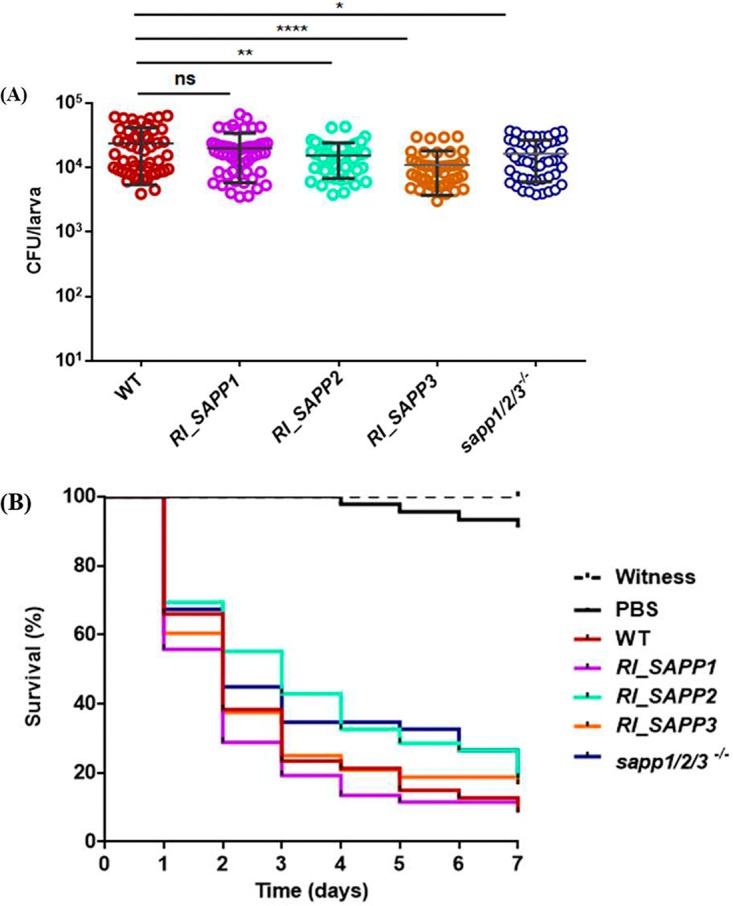
Virulence of C. parapsilosis wild-type and *SAPP* mutant strains in G. mellonella. (A) Fungal burden of G. mellonella larvae infected with the wild-type strain or a *SAPP* mutant strain. Larvae were incubated at 30°C for 24 h. (B) Survival curves of G. mellonella infected with C. parapsilosis wild-type strain and *sapp* mutant strains. The infected larvae were incubated at 30°C for 7 days. Four individual experiments were performed using at least five larvae per round for CFU counting. Two individual experiments were performed using 24 larvae per round for the survival assay. *, *P* < 0.05; **, *P* < 0.01; ****, *P* < 0.0001.

CFU recovery data show that *RI_SAPP1* produced CFU numbers similar to those seen with wild-type C. parapsilosis in G. mellonella larvae (Fig. 12A). In contrast, the virulence of the other mutants was attenuated compared to that of the parental strain.

Overall, larvae infected with wild-type and mutant strains showed no significant difference in survival after the 7 days of infection (Fig. 12B).

## DISCUSSION

Aspartyl proteases are present in a diverse range of microorganisms and play a crucial role in nutrition acquisition and pathogenesis. The presence of aspartyl proteases in pathogenic *Candida* species and their absence in nonpathogenic fungal species such as Saccharomyces cerevisiae suggests their role in pathogenesis. Previously, we showed that C. parapsilosis *Δ*/*Δsapp1a*, *Δ*/*Δsapp1b*, and *Δ*/*Δsapp1a-*/*Δ*/*Δsapp1b* deletion mutant strains are less virulent than the wild-type strain, demonstrating that Sapp1p plays a role in pathogenesis regulation. To date, the roles of *SAPP2* and *SAPP3* in C. parapsilosis virulence have not been investigated. Therefore, in the present study, we aimed to delineate their roles in pathogenicity using a secreted aspartyl protease-deficient strain (*sapp1*/*2*/*3^−^*^/^*^−^*) and mutant strains that express each *SAPP* gene individually under the control of a constitutive promoter (*pCaTDH3*).

In C. albicans, secreted proteins play an important role in morphology and biofilm formation (33–36). Hence, we first determined the corresponding effects of SAPP proteins in C. parapsilosis. In contrast to C. albicans, C. parapsilosis SAPP proteins do not affect either of these properties. SapII, SapV, and SapVI were previously reported to play a role in tissue adhesion C. albicans in addition to their role in biofilm formation (37). Furthermore, C. albicans Sap1p, Sap2p, Sap3p, and Sap9p were previously reported to be involved in adherence to epithelial cells (10, 38, 39). In the present study, we showed that Sapp1p, Sapp2p, and Sapp3p in C. parapsilosis also contribute to adhesion, although possibly to differing degrees.

As shown by examining the effect of cell wall-perturbing agents, disruption of the *SAPP* genes did not affect the mutant strain’s survival, indicating that C. parapsilosis aspartyl proteases do not influence the species’ fitness and viability.

On the other hand, disruption of *SAPP1* and *SAPP2* but not *SAPP3* resulted in serum sensitivity. These results suggest that only the former two proteases are required for serum survival in this species. This observation is consistent with a previous finding according to which enhanced Sapp1p production was detected in C. parapsilosis cells in the presence of serum albumin (28).

Pathogenic fungi have been previously reported to overcome the fungicidal effects of human serum via actively secreting aspartyl proteases to neutralize proteins with antimicrobial effects (15, 40). For instance, complement proteins have diverse functions that include opsonization of microbes to facilitate phagocytosis, activation of cellular responses, initiation of inflammation, and direct lysis of microbial cells (41, 42). The protective effects of Sapp1p and Sapp2p mentioned above might be the result of their ability to cleave complement components. Therefore, we further aimed to examine the complement cleavage activity of purified Sapp1p and Sapp2p proteins. Complement component 3 (C3) plays a central role in all three complement pathways. Following its cleavage by C3 convertase, the resulting C3b fragment forms the C5 convertases that are necessary for the progression of the complement cascade. Our results suggest that C. parapsilosis is able to escape such complement-mediated attacks through the activity of its secreted aspartyl proteases, as both Sapp1p and Sapp2p are able to efficiently degrade the active complement C3b and C4b fragments required for convertase functioning and opsonization, similarly to the degradation and thus inactivation in the host mediated by serine protease factor I, a complement control protein (CCC).

FH and FHL-1 inhibit complement activation in the host but also do so when sequestered from serum by pathogenic microbes as an immune escape mechanism. FH and FHL-1 bind to microbial ligands through specific domains that are partially conserved among other members of the FH protein family, i.e., the FHR proteins (43, 44). FHRs were also reported previously to be involved in complement cascade regulation, although this is a controversial issue (43, 45, 46). FHR-1 was reported to inhibit C5 and the terminal pathway, whereas FHR-2 inhibits the alternative pathway and activation of the terminal pathway. FHR-5 displays weak cofactor activity and inhibits the C3 convertase and was recently reported to inhibit C5 conversion (47–51). On the other hand, FHR-1, FHR-4, and FHR-5 were shown to support alternative pathway activation at the C3 level by binding C3b and allowing the formation of the C3 convertase (52–54). Thus, FHRs—due to the presence of conserved domains—may competitively inhibit FH/FHL-1 binding to microbes and enhance opsonization (50, 55). According to our results, neither FHL-1 nor FHR-1 is cleaved by C. parapsilosis Sapp1p or Sapp2. Furthermore, a difference in substrate preference is also evident, as Sapp2p, but not Sapp1p, is able to cleave FHR-5. The cleavage of FHR-5 but not FHL-1 and FHR-1 suggests that Sapp2p presumably cleaves at locations near complement control protein (CCP) domains 3, 4, 5, 6, and 7, which are absent in FHL-1 and FHR-1 but present in FHR-5 and FH, although further studies are needed to confirm this hypothesis. These data suggest that the secreted aspartyl proteases of this species show a substrate preference for complement proteins involved in activation of the cascade, rather than for complement control proteins (e.g., factor H family proteins).

C. albicans attachment and subsequent colonization are necessary to induce inflammatory responses in epithelial cells (56). Activation of epithelial cells also shapes the responses of monocytes, macrophages, and other immune cells during a fungal infection. Professional antigen-presenting cells such as macrophages connect the innate and adaptive arms of the host’s immune responses by processing and presenting antigens to other effector cells and actively eliminating pathogens. Thus, we next examined if disruption of any of the C. parapsilosis *SAPP* genes would have an effect on macrophage activity. Our results indicate that human PBMC-DMs were able to phagocytose and eliminate *sapp1*/*2*/*3^−^*^/^*^−^* and *RI_SAPP3* cells more efficiently than the wild-type and *RI_SAPP1* or *RI_SAPP2* strains.

The aspartyl proteases of C. albicans induce proinflammatory cytokine responses to differing degrees. For instance, SapI, SapII, and SapVI significantly induce IL-1β, TNF-α, and IL-6 production, while SapIII is able to stimulate IL-1β and TNF-α secretion (57). Besides inducing low levels of host cell damage, the *sapp1*/*2*/*3^−^*^/^*^−^* and *RI_SAPP3* strains also induced lower levels of proinflammatory cytokines (IL-1β, IL-6, and IL-8) than the parental and *RI_SAPP1* or *RI_SAPP2* strains. These results, together with the data gathered from G. mellonella infection (an invertebrate model commonly applied to mimic basic cellular and humoral mammal-like immune responses *in vivo* [58]), further suggest differences in the contribution of C. parapsilosis Sapp proteins to virulence.

In conclusion, we demonstrated in the present study that C. parapsilosis Sapp proteins did not affect formation of pseudohyphae or biofilm. However, Sapp1p and Sapp2p play roles in adhesion to epithelial cells and in host cell damage and might promote survival within macrophages. Sapp-mediated cleavage of complement proteins also suggests that C. parapsilosis might also interfere with human complement attack. In summary, Sapp1p and Sapp2p, but not Sapp3p, are the major and fully functional aspartyl proteases in C. parapsilosis that actively affect the species’ pathogenicity.

## MATERIALS AND METHODS

### Strains and growth conditions.

The strains used in the present study and their abbreviations are listed in Table S1 in the supplemental material. Strains were cultured overnight in YPD broth at 30°C, with shaking. Cells from overnight cultures were collected by centrifugation and washed twice with sterile 1× PBS (phosphate-buffered saline), and the number of cells was adjusted as indicated in descriptions of the respective experiments. For growth assays and gene expression studies, the wild-type and mutant strains were cultivated in YCB (yeast carbon base) medium supplemented with 0.2% BSA (bovine serum albumin) at 30°C. Escherichia coli DH5α was grown in LB (Luria-Bertani broth) or on LB plates supplemented with ampicillin (0.1 mg/ml) for plasmid construction and propagation.

10.1128/mSphere.00484-19.8 TABLE S1 List of strains used in the present study. Download Table S1, DOCX file, 0.02 MB. Copyright © 2019 Singh et al. 2019 Singh et al. This content is distributed under the terms of the Creative Commons Attribution 4.0 International license.

### Generation of C. parapsilosis secreted aspartyl protease mutant strains.

*sapp*1/2/3^−/−^ mutants were generated as described previously (15) with minor modifications. Briefly, ∼500-bp upstream and downstream regions of *SAPP2* and *SAPP3* were PCR amplified and cloned in the pSFS2a plasmid with a recyclable NAT cassette. Further, the *SAPP2* deletion cassette was introduced in the *ΔΔsapp1a ΔΔsapp1b* deletion mutant strains to generate *ΔΔsapp1a ΔΔsapp1b ΔΔsapp2* mutants. Finally, the *SAPP3* deletion cassette was generated similarly to *SAPP2*, and *ΔΔsapp1a ΔΔsapp1b ΔΔsapp2* mutant strains were transformed with the construct to generate the *sapp1*/*2*/*3*^−/−^ strain.

Mutant strains expressing the individual *SAPP* genes were generated using the *SAPP1*-*SAPP2*-*SAPP3* (*sapp1*/*2*/*3^−^*^/^*^−^*) null mutant strain. Solely *SAPP1*-, *SAPP2*-, and *SAPP3*-expressing mutants were established using a replacement cassette targeting the Neut5l locus and containing the respective *SAPP* open reading frames (ORFs) under the control of the *CaTDH3* constitutive promoter. In each case, nourseothricin was used as a selection marker. C. parapsilosis strains were transformed by electroporation as described previously (59). The transformants were confirmed by colony PCR and Southern blot analysis.

### Gene expression studies.

Total RNA was isolated from C. parapsilosis wild-type cells grown in YCB plus 0.2% medium for 48 h using a RiboPure RNA purification kit according to the manufacturer’s instructions. A 500-ng volume of RNA was subjected to reverse transcription using a RevertAid first-strand cDNA synthesis kit according to the protocol provided by the manufacturer. Quantitative PCR (qPCR) was performed using the primers listed in Table S2. The amplification conditions were as follows: one cycle of denaturation for 3 min at 95°C; denaturation at 95°C for 10 s; 49 cycles of annealing at 60°C for 30 s, and elongation at 65°C for 30 s; with a final extension step at 72°C for 30 s. *TUB4* was used as an internal control.

10.1128/mSphere.00484-19.9 TABLE S2 Primers used for *SAPP* gene expression. Download Table S2, DOCX file, 0.02 MB. Copyright © 2019 Singh et al. 2019 Singh et al. This content is distributed under the terms of the Creative Commons Attribution 4.0 International license.

10.1128/mSphere.00484-19.10 TABLE S3 Phenotype screening of *sapp1*/*2*/*3^−^*^/^*^−^* strain. Growth of the deletion collection was determined under different stress conditions designed to identify the YPD used as a base media (except when YCB medium was used). Download Table S3, DOCX file, 0.02 MB. Copyright © 2019 Singh et al. 2019 Singh et al. This content is distributed under the terms of the Creative Commons Attribution 4.0 International license.

### Functional studies of the generated mutant strains.

Functional studies of the generated strains were performed as described previously (55, 60). Detailed descriptions of growth analysis and assays required to determine extracellular protease activity, formation of pseudohyphae, biofilm formation, adhesion capabilities, stress response, serum sensitivity, phagocytosis, and yeast cell killing are available in the supplemental material.

### Human epithelial cell lines (TR146).

The human buccal epithelial squamous carcinoma TR146 cell line was kindly provided by Julian Naglik, Kings College London, United Kingdom, and cultured as described previously (61).

### Isolation and differentiation of PBMCs.

Human peripheral blood mononuclear cells (PBMCs) were isolated from buffy coats of healthy donors by Ficoll Paque Plus (GE Healthcare) density gradient centrifugation and used to produce macrophages as described previously (62).

### Cell damage (lactate dehydrogenase activity) assay.

LDH activity in cell culture supernatants was measured at 24 or 48 h of postinfection using a cytotoxicity detection kit (LDH; Roche) according to the manufacturer’s instructions. Macrophages were stimulated with C. parapsilosis wild-type, *RI_SAPP*, and *sapp1*/*2*/*3^−^*^/^*^−^* cells at a ratio of 1:5 (host cell/*Candida* cell) for 24 or 48 h or left untreated. During analysis, the values corresponding to the levels of LDH activity measured in cultures containing yeast cells alone were subtracted from the values measured in stimulated samples. Experiments were performed with PBMC-DMs derived from six independent donors in triplicate experiments.

### Phagolysosome fusion.

Fusion of phagosomes-lysosomes after infection was assayed as described previously (63). Both the phagocytosis and phagolysosome fusion assays were performed with PBMC-DMs derived from five independent donors.

### Cytokine measurements.

PBMC-DMs were infected with 5 × 10^5^ fungal cells, and supernatant was collected after 24 h of incubation. Then, the concentrations of secreted IL-1β, IL-6, IL-8, and TNF-α in cell culture supernatants were determined by the use of commercial enzyme-linked immunosorbent assay (ELISA) kits (R&D Systems) according to the manufacturer’s instructions. The experiments were performed with PBMC-DMs derived from the blood of at least five independent donors.

### Purification of Sapp1p and Sapp2p.

Sapp1p and Sapp2p were purified as described previously (64, 65). Proteins were stored at –80°C until use.

### Cleavage activity.

The proteolytic activity of purified Sapp1p and Sapp2p (1 μg each) was assayed by incubating them with purified human complement proteins C3b, C4b, and factor H (FH) (Merck) or with recombinant factor H-like protein 1 (FHL-1) (expressed and purified as described previously) (66) or FHR-1 or FHR-5 (Novoprotein) for 3 h or 15 h at 37°C. Aliquots were taken at the indicated time points, separated by SDS-PAGE, and analyzed by Western blotting. C3b was identified by the use of polyclonal goat anti-human C3 (Calbiochem, Quidel) in combination with a horseradish peroxidase (HRP)-conjugated goat antibody (DAKOCytomation). C4b was detected with a monoclonal anti-C4c antibody (Quidel) and with HRP-conjugated goat anti-mouse Ig (Dako). To detect cleavage of FH, FHL-1, FHR-1, and FHR-5, polyclonal goat anti-FH (Calbiochem, Merck), mouse monoclonal anti-FH (A254; from Quidel), and polyclonal goat anti-FHR-5 (R&D System) and the corresponding HRP-conjugated secondary antibodies rabbit anti-goat Ig and goat anti-mouse Ig (Dako) were used. In addition, cleavage of C3b and C4b by the natural, complement-specific protease factor I in the presence of the cofactors factor H and C4BP (Hyphen Biomed) was assayed to compare with the cleavage patterns generated by the Sapp proteases.

### Galleria mellonella infection.

Galleria mellonella larvae (TruLarv) (0.20 to 0.35 g) were purchased from Biosystems Technology Ltd., Exeter, United Kingdom. Upon arrival, the larvae were handled and injected with wild-type or mutant strains as described previously (55).

For CFU determination, larvae (0.25 to 0.30 g) were infected with 10^5^ *Candida* cells/10 μl and sacrificed at 24 h postinfection and the fungal load of each individual larva was determined. Briefly, each larva was homogenized in 5 ml of PBS. The homogenate was plated on YPD plates and incubated at 30°C for 2 days, and the colonies were counted.

To monitor survival, the larvae used in the infection experiments were infected with 10^6^ *Candida* cells/10 μl and kept at 30°C for 7 days and larval death was monitored every day. Groups of 5 larvae were used per strain with four experimental replicates for CFU and 24 larvae per strain with two experimental replicates for survival.

### Ethics statement.

For PBMC isolation, blood was collected from healthy individuals. The Institutional Human Medical Biological Research Ethics Committee of the University of Szeged gave approval for the procedure and the respective consent documents. Healthy individuals provided written informed consent. The experiments were performed in accordance with the guidelines and regulations of the Ethics Committee of the University of Szeged, and the experimental protocols were approved by the same institutional committee.

### Statistical analysis.

Unpaired *t* tests were used to determine differences between the group results determined by adhesion assay, LDH assay, phagocytosis assay, killing assay, cytokine analysis, and CFU data analysis. Mantel-Cox (log rank) tests were used for evaluation of survival data. Differences were considered statistically significant at *P* values of ≤0.05 (*, *P* ≤ 0.05; **, *P* ≤ 0.01; ***, *P* ≤ 0.001).
