# Supplementary material for: Functional Characterization of Secreted Aspartyl Proteases in Candida parapsilosis
Source: mSphere. 2019 Aug 21;4(4):e00484-19. doi: 10.1128/mSphere.00484-19 (PMC6706470; doi:10.1128/mSphere.00484-19)
Supplement: TABLE S1 [file mSphere.00484-19-st001.docx]

| **Strain name** | **Parent strain** | **CpAR gene ID** | **Genotype** | **Reference** |
| --- | --- | --- | --- | --- |
| GA1 |  |  |  | Gácser et al., 2007* |
| *sapp1/2/3^−/−^* | GA1 |  | *Δ/Δsapp1a-Δ/Δsapp1b- Δ/Δsapp2-Δ/Δsapp3* |  |
| *RI_SAPP1* | *sapp1/2/3^−/−^* | CPAR2_102410 | *Δ/Δsapp1a-Δ/Δsapp1b- Δ/Δsapp2-Δ/Δsapp3, Cpneut5l::CaTDH3-SAPP1-NAT/CpNEUT5L* | This study |
| *RI_SAPP2* | *sapp1/2/3^−/−^* | CPAR2_102580 | *Δ/Δsapp1a-Δ/Δsapp1b- Δ/Δsapp2-Δ/Δsapp3, Cpneut5l::CaTDH3-SAPP2-NAT/CpNEUT5L* | This study |
| *RI_SAPP3* | *sapp1/2/3^−/−^* | CPAR2_102420 | *Δ/Δsapp1a-Δ/Δsapp1b- Δ/Δsapp2-Δ/Δsapp3, Cpneut5l::CaTDH3-SAPP3-NAT/CpNEUT5L* | This study |

*Gácser A, Trofa D, Schäfer W, Nosanchuk JD. 2007. Targeted gene deletion in *Candida parapsilosis* demonstrates the role of secreted lipase in virulence. J. Clin. Investig. 117:3049–3058. <https://doi.org/10.1172/JCI32294>.
