# Supplementary material for: Functional Characterization of Secreted Aspartyl Proteases in Candida parapsilosis
Source: mSphere. 2019 Aug 21;4(4):e00484-19. doi: 10.1128/mSphere.00484-19 (PMC6706470; doi:10.1128/mSphere.00484-19)
Supplement: TABLE S2 [file mSphere.00484-19-st002.docx]

| **Primer** | **Sequence** |
| --- | --- |
| *SAPP1* (F) | 5’-ACTGGACAACAAATTGCAGATG-3’ |
| *SAPP1* (R) | 5’-TAAACTGCTTCATTGCTGGTGT-3’ |
| *SAPP2* (F) | 5’-GTCATATGGGGGATTTGCAC-3’ |
| *SAPP2* (R) | 5’-CGCTTTGCTGATGTTACCAG-3’ |
| *SAPP3* (F) | 5’-CTGGGTCATTGATGCAAATTC-3’ |
| *SAPP3* (R) | 5’-AGGTTGAGGTGTCTGGATCG-3’ |
