# Supplementary material for: Functional Characterization of Secreted Aspartyl Proteases in Candida parapsilosis
Source: mSphere. 2019 Aug 21;4(4):e00484-19. doi: 10.1128/mSphere.00484-19 (PMC6706470; doi:10.1128/mSphere.00484-19)
Supplement: TABLE S3 [file mSphere.00484-19-st003.docx]

| **Condition** | **Reagent** | **Applied concentration/amount** |
| --- | --- | --- |
| **pH** | 5,6,7 and 8 |  |
| **Osmotic stress** | Glycerol | 8%, 10%, 12% |
|  | Sorbitol | 1M, 1.5M |
|  | Nacl | 1M, 1.5M |
| **Oxidative stress** | H_2_O_2_ | 10mM |
|  | CdSo_4_ | 0.05mM |
|  | Menadione | 0.2mM |
| **Cell wall perturbant** | Caffeine | 10mM |
|  | Calcoflour white | 10, 25,50µg/ml |
|  | Congo Red | 10, 25,50, 75, 100µg/ml |
| **Membrane perturbant** | SDS | 0.02%, 0.04% |
|  | CuSo_4_ | 4mM |
|  | CuCl_2_ | 4mM |
| **Metal ion starvation** | EDTA | 0.25mM |
|  | BPS | 150µg/ml |
